# Jaundice, hepatosplenomegaly, and portal lymphadenopathy in a middle‐aged female: Is it lymphoma?

**DOI:** 10.1002/jgh3.12211

**Published:** 2019-06-24

**Authors:** Alexander T Elford, Jeremy P Dwyer, Scott B Fanning

**Affiliations:** ^1^ Department of General medicine Royal Hobart Hospital Hobart Tasmania Australia; ^2^ School of Medicine University of Tasmania Hobart Tasmania Australia; ^3^ Department of Gastroenterology and Hepatology Launceston General Hospital Launceston Tasmania Australia

**Keywords:** lymphadenopathy, lymphoma, primary biliary cholangitis

## Abstract

Primary biliary cholangitis is a rare liver disease which often progresses to cirrhosis. It can be difficult to diagnose as patients are often asymptomatic initially or merely complain of fatigue or pruritus. We describe the case of a 56‐year‐old female who presented with a 2‐month history of painless jaundice and constitutional symptoms. Computed tomography scan showed massive hepatosplenomegaly with abdominal lymphadenopathy. Liver biopsy and a strongly positive antimitochondrial antibody titer confirmed the diagnosis of primary biliary cholangitis.

## Graphical abstract

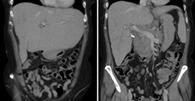

## Introduction

Primary biliary cholangitis is a rare liver disorder which predominantly presents in middle aged females and often leads to cirrhosis. When diagnosed, patients are often asymptomatic or only complain of fatigue or pruritus. We report a case of primary biliary cholangitis which was highly suspicious for lymphoma with B‐symptoms and massive hepatosplenomegaly as the presenting complaints.

## Case report

A 56‐year‐old female presented with a 2‐month history of painless jaundice, pruritus, weight loss, and night sweats. Past medical history included laparoscopic cholecystectomy for cholelithiasis 2 years prior, at which time an enlarged peripancreatic lymph node was noted. The peripancreatic lymph node measured 25 mm on computed tomography (CT), and there was no interval change on surveillance imaging 1 year postcholecystectomy. The patient took no regular medications, consumed minimal alcohol, and was a nonsmoker. Family history was noncontributory.

**Figure 1:**
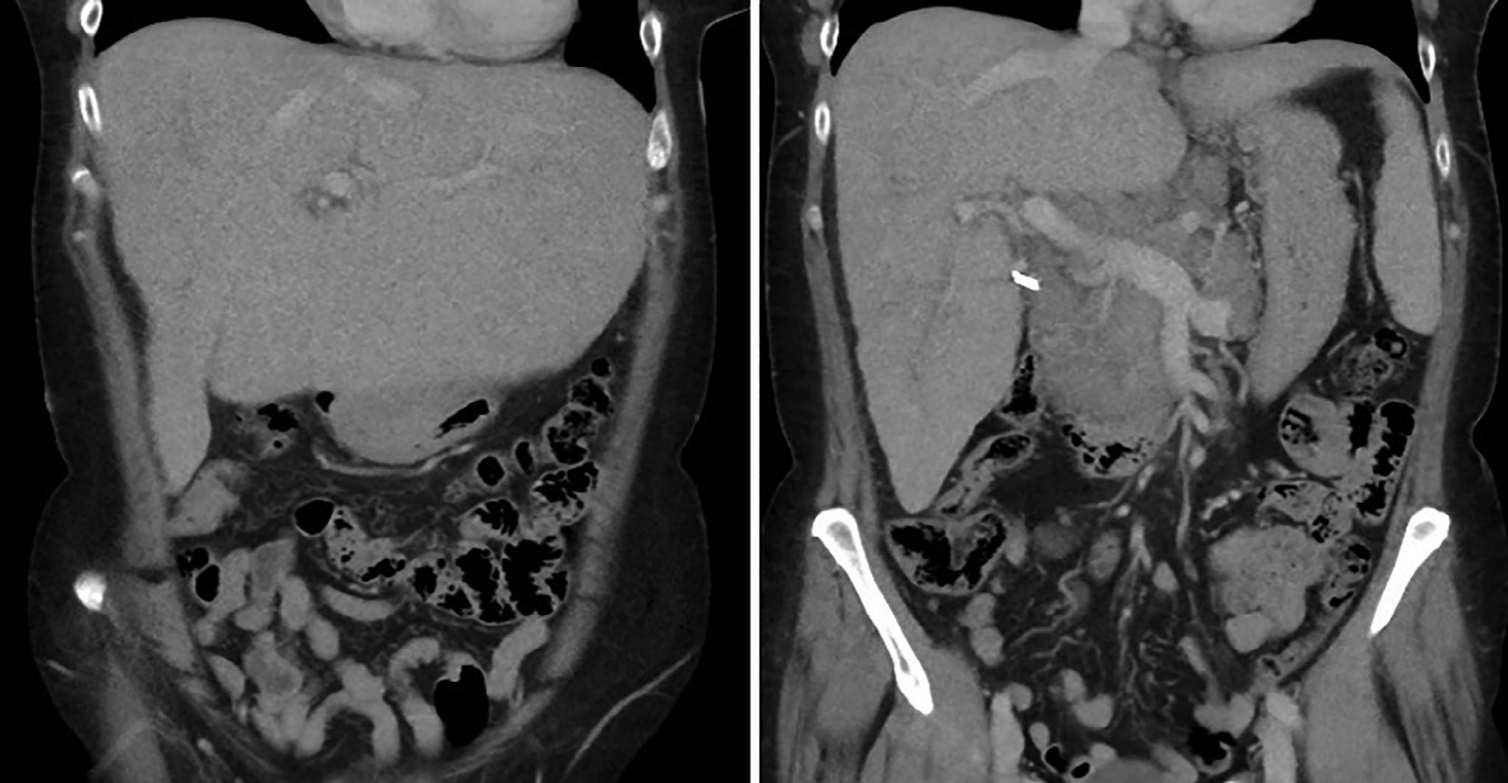
Computed tomography abdomen‐pelvis demonstrated hepatomegaly with a craniocaudal span of 23 cm, splenomegaly measuring 14 cm, and a soft tissue mass at the porta hepatis consistent with confluent lymphadenopathy with several other enlarged lymph nodes in the region of the coeliac axis and upper para‐aortic region measuring up to 35 mm.

Physical examination demonstrated jaundice and hepatomegaly. Liver function tests (LFTs) were deranged, with bilirubin = 107 μmol/L (normal range < 21 μmol/L), alanine aminotransferase = 113 U/L (normal range < 33 U/L), gamma‐glutamyl transferase (GGT) = 1333 U/L (normal range < 40 U/L), and alkaline phosphatase = 1461 U/L (30–110 U/L). Lipase was normal at 40 U/L (normal range = 13–60 U/L). Lactate dehydrogenase was normal at 104 U/L (normal range = 125–250 U/L); however, both CA19‐9 = 219 U/mL (normal range < 39 U/mL) and Beta 2 microglobulin = 4.36 mg/L (normal range = 0.8–2.2 mg/L) were elevated. The patient subsequently had a CT abdomen‐pelvis (Fig. 1), which demonstrated hepatomegaly with a craniocaudal span of 23 cm, splenomegaly measuring 14 cm, and a soft tissue mass at the porta hepatis consistent with confluent lymphadenopathy with several other enlarged lymph nodes in the region of the coeliac axis and upper para‐aortic region measuring up to 35 mm.

Magnetic resonance cholangiopancreatography demonstrated displacement and mild narrowing of the common bile duct by the large periportal nodal mass and variant biliary anatomy with the cystic duct joining the right hepatic duct. In view of the significant lymphadenopathy, endoscopic ultrasound (EUS) with fine‐needle aspiration (FNA) of the periportal lymph nodes (measuring 44 mm on EUS) was performed to evaluate for lymphoma or metastases. Cytology from EUS‐FNA demonstrated a normal lymphoid population with no evidence of malignancy. Liver biopsy was then performed, which demonstrated lobular inflammation with bile ductular reaction and distortion of the liver lobules and portal triads by fibrosis suggesting macronodular cirrhosis. Serology was strongly positive for antimitochondrial antibody (titer > 1:2560), consistent with a diagnosis of primary biliary cholangitis (PBC) with associated cirrhosis.

## Discussion

This case highlights that PBC may sometimes present with hepatosplenomegaly and lymphadenopathy. Abdominal lymphadenopathy is not uncommon in patients with chronic liver disease. In a series of 227 patients, 19% of patients with chronic liver disease were found to have abdominal lymphadenopathy on ultrasound.^1^ Lymphadenopathy is more common in autoimmune liver diseases, of which PBC has the highest prevalence (33%).^1^ Other studies have shown that 88 and 64% of PBC patients had lymphadenopathy reported on CT^2^ and magnetic resonance imaging,^3^ respectively. We conclude that, once lymphoma and malignancy are excluded, PBC should be considered a differential diagnosis in patients with deranged LFTs, hepatosplenomegaly, and lymphadenopathy.
